# Cutaneous manifestations of pediatric Langerhans cell histiocytosis: A single-center case series from 2019 to 2024

**DOI:** 10.1016/j.jdcr.2026.04.045

**Published:** 2026-04-30

**Authors:** Ma. Veronica Pia N. Arevalo, Amanda T. Chung, Krisha K. Lim, Vinz Troy M. Solanoy, Christine Bernadette O. Lo, Juan Paolo B. Torres, Kaira Monique G. Osmeña, Kathleen Stephanie D. Wahing, Val Constantine S. Cua, Carmela Augusta F. Dayrit-Castro

**Affiliations:** a Department of Dermatology, University of the Philippines – Philippine General Hospital, Manila, Philippines; b Hematology and Oncology, Department of Pediatrics, University of the Philippines – Philippine General Hospital, Manila, Philippines

**Keywords:** biopsy, Hand-Schüller-Christian disease, histiocytosis X, Langerhans cell histiocytosis, Letterer-Siwe disease, nail diseases, scalp dermatoses

## Introduction

Histiocytic disorders are characterized by the pathologic proliferation of clonal cells derived from monocyte, macrophage, or dendritic cell lines.^1^ In Langerhans cell histiocytosis (LCH), historically known as histiocytosis X, CD1a-positive immature dendritic cells proliferate and infiltrate various organ systems.^2^ It is the most common histiocytic disorder, and it is more prevalent among children than adults.^1^^,^^2^ The disease may be classified as either single-system single-site, multi-site, or multisystem type (MS),^2^ with the bones, skin, and pituitary gland being the most commonly involved organs. The liver, spleen, and bone marrow signify risk organ involvement.^1^^,^^3^ Cutaneous involvement is often indicative of multisystemic disease, with only 2% accounting for isolated cutaneous LCH.^1^ Skin lesions in LCH may mimic other skin disorders, with seborrheic dermatitis being the most common, and nail involvement is a rare but notable finding. We describe ten pediatric patients seen at the dermatology department of a tertiary center in the Philippines from 2019 to 2024 with diverse skin and nail manifestations of LCH. Their clinical profiles are summarized in Table I. This case series aims to foster morphologic pattern recognition, to emphasize a systematic and thorough approach to skin examination, and to reinforce awareness of rare cutaneous presentations of LCH, ultimately supporting earlier detection in clinical practice.

**Table I:** Clinical profiles of study patients

Patient	Sex	Age of symptom onset	Age at diagnosis	Cutaneous manifestations	Organs involved	Risk stratification	Treatment	Outcome
1	M	Early weeks of life	3 y	Seborrheic scalp Hemorrhagic papules on the face Globe dystopia Longitudinal melanonychia	Bone Skin Lymph nodes Cranium	Multi-system LCH with risk organ involvement (liver)	LCH-IV stratum I protocol	Ongoing treatment
2	M	11 mo	1 y and 1 mo	Erosive erythematous plaques on axillary, inguinal, and intergluteal folds (mimicking napkin dermatitis) Crusted papules on palms and soles Petechiae on trunk (predominantly on flexural folds) Splinter hemorrhages on the fingernails and toenails	Bone Skin	Multi-system LCH without risk organ involvement	JLSG-96 protocol	Ongoing treatment
3	M	1 y	2 y	Seborrheic scalp with nonadherent white and greasy yellow scales Solitary parietal scalp nodule	Bone Skin	Multi-system LCH without risk organ involvement	s/p LCH IV stratum I (2022-2023) s/p relapse regimen (2023-2024) Ongoing JLSG-96 protocol	Recurrence
4	M	1 y	1 y and 7 mo	Rose-colored papules on the trunk and flexural areas Seborrheic papules on the scalp, forehead, and glabella Trachyonychia and splinter hemorrhages on bilateral hands	Skin Liver Spleen	Multi-system LCH with risk organ involvement (liver, spleen)	s/p JLSG-96 protocol Currently on LCH IV Stratum I	Ongoing treatment
5	F	5 mo	1 y and 6 mo	Scalp erythema with greasy yellow scales Skin-colored and rose-colored papules on the trunk, inguinal folds, and genitalia Orbital mass, globe dystopia	Skin Bone	Multi-system LCH without risk organ involvement	LCH-IV stratum I protocol	Completed treatment Ongoing surveillance
6	M	3 y	5 y	Nonhealing scalp ulcer Subungual hemorrhages Onycholysis Subungual hyperkeratosis Fleshy vascular subungual tumors	CNS (pituitary) Skin	Multi-system LCH without risk organ involvement	Vincristine, cytarabine, prednisolone (JLSG-96 protocol)	Completed treatment Ongoing surveillance
7	M	1 y and 7 mo	2 y	Seborrheic papules and plaques on the scalp Rose-colored and flesh-colored papules on the trunk Eczematous patches on the neck, right axilla, bilateral inguinal creases, and perianal areas	Skin Bone	Multi-system LCH without risk organ involvement	LCH-IV stratum I protocol	Ongoing treatment
8	M	1 y	2 y	Salmon-colored papules with white scales on the scalp Erythematous patches on the inguinal region Globe dystopia	Skin Bone	Multi-system LCH without risk organ involvement	LCH-IV stratum I protocol s/p LCH IV stratum I (2024) Ongoing JLSG-96 protocol	Recurrence
9	F	1 y	3 y	Erythematous scaly papules and excoriations on the scalp, trunk, and extremities Trachyonychia Periungual swelling, erythema, and pustules Icteric sclerae, gingival hyperplasia, jaundice	Skin Liver	Multi-system LCH with risk organ involvement (liver)	LCH-IV stratum II protocol (without vincristine due to elevated transaminases)	Ongoing treatment
10	F	1 y and 2 mo	1 y and 6 mo	Hemorrhagic macules and papules on the scalp, palms, and back Jaundice	Skin Bone Liver Spleen	Multi-system LCH with risk organ involvement (liver, spleen)	LCH-IV stratum I protocol	Completed treatment Ongoing surveillance

*LCH*, Langerhans cell histiocytosis.

## Case series

### Patient no. 1

RA, a 3-year-old male, presented with multiple erythematous scaly papules on the forehead and scalp since infancy, and later developed proptosis of the left eye and multiple cervical lymphadenopathies at 2 years old. Initially lost to follow up, he later underwent a cranial computed tomography scan, which showed intracranial masses, and a skeletal survey which showed lytic changes on the cranium. A 3.5 mm punch biopsy on the scalp revealed dense lichenoid infiltrate composed of histiocytes, some with reniform nuclei which stained positive for S100, CD1a, and CD207, confirming the diagnosis of LCH.

### Patient no. 2

MC, a 1-year-old male, presented with erythematous papules and plaques with yellow crusts on the hypogastric area, back, palms, soles, and intertriginous areas. In addition, he also presented with left thigh swelling and splinter hemorrhages on his fingernails. Lesions were unresponsive to topical corticosteroids and antifungals. A 4 mm skin punch biopsy of an erythematous papule on the hypogastric area showed histiocytes with reniform nuclei with strong cytoplasmic and membranous CD1a and CD207 staining patterns, consistent with LCH.

### Patient no. 3

JL, a 4-year-old male, is a known case of multi-system LCH with skin and bone involvement diagnosed at the age of 2. He underwent LCH-IV Stratum I protocol standard chemotherapy regimen for 1 year and relapse regimen composed of vincristine, cytarabine, and prednisone for 8 months; a year after treatment, he developed new-onset asymptomatic scaly yellowish-brown papules on the right parietal scalp with no involvement of the face and body. A repeat lesional 4 mm skin punch biopsy revealed subacute spongiotic dermatitis with reniform cells with strong cytoplasmic CD1a and CD207 staining patterns, confirming relapse in this case. Currently, the patient is ongoing JLSG-96 protocol.

### Patient no. 4

KT is an 18-month-old male with a 6-month history of multiple erythematous papules and pustules coalescing into greasy plaques on the scalp and rose-colored papules on the face, trunk, and suprapubic area, which persisted despite 2 courses of oral antibiotics. Two months prior to admission, he developed oral ulcers, undocumented febrile episodes, and splinter hemorrhages on the nails. Workup revealed severe anemia and hepatosplenomegaly. Skeletal survey was unremarkable. A 4-mm skin punch biopsy of a papule on the nape revealed dense perivascular collections of histiocytes in the papillary dermis, with CD207 staining clusters of reniform cells, consistent with LCH.

### Patient no. 5

DV, at 5 months old, developed pruritic, erythematous, seborrheic papules on the scalp, ears, axillae, back, and genitals, unresolved with topical antifungals. Four months later, she developed ptosis of the left eyelid due to an enlarging superolateral orbital mass but only sought consultation at 14 months old. Lesional skin punch biopsy on the back showed dense band-like histiocytic infiltrates with reniform nuclei, which stained positive for CD1a. Skeletal survey showed osteolytic lesions on the left frontal bone and superior orbital rim. Holoabdominal ultrasound was normal. She was started on chemotherapy for LCH, with no recurrence 1 year from completion of treatment.

### Patient no. 6

NS is a 5-year-old male who presented with onycholysis and longitudinal red streaks on the fingernails and toenails at the age of 3, followed months later by a nonhealing ulcer on the left parietal scalp. He had no other cutaneous lesions but also exhibited esotropia and polyuria. Workup revealed pituitary enlargement, central diabetes insipidus, and hypothyroidism. Initial scalp and nail bed punch biopsies showed inflamed granulation tissue and a focal acantholytic suprabasilar split, respectively. He was managed supportively with intralesional and topical steroids but was lost to follow-up. A year later, he returned with an enlarging scalp ulcer; excision biopsy revealed clusters of reniform cells in the papillary dermis, staining positive for CD1a and CD207. Holoabdominal ultrasound and skeletal survey were unremarkable. A diagnosis of multi-system LCH was made, and chemotherapy was initiated, leading to the resolution of nail changes and no recurrence of the scalp ulcer.

### Patient no. 7

PM is a 2-year-old male who presented with a 5-month history of multiple seborrheic papules and plaques with thick adherent greasy scales on the scalp which bleed easily upon manipulation. He also had multiple rose- and flesh-colored papules on the chest, abdomen, and back. There were multiple fairly-defined erythematous eczematous patches on the neck, axillae, inguinal creases, and perianal area. A 3 mm skin punch biopsy of a scalp papule revealed a dense lichenoid infiltrate of predominantly histiocytes in the superficial dermis, which stained positive for S100, CD1a, and CD207. Cranial computed tomography scan revealed multiple osteolytic lesions involving the left sphenoid bone, right parietal bone, left occipital bone, right alveolar process, and right maxilla.

### Patient no. 8

CS is a 2-year-old male who presented with an 8-month history of left eye redness, tearing, and discharge, progressing to proptosis. Cranial computed tomography revealed heterogeneously enhancing soft tissue lesions with lytic destruction of the calvarium and skull base. Additional findings included persistent nasal discharge, occipital and temporal masses, and scaly, salmon-colored papules on the scalp with erythema in the diaper area. Lesional skin biopsy showed dense lichenoid infiltrates in the papillary dermis, composed of lymphocytes, eosinophils, neutrophils, and histiocytes with reniform nuclei. CD1a stain highlighted the Langerhans cells, confirming a diagnosis of LCH.

### Patient no. 9

EL is a 3-year-old female who presented with a 2-year history of abdominal pain and distention from hepatosplenomegaly, followed by pruritic erythematous papules on the abdomen, back, and extremities. Three months later, the patient developed jaundice and gingival swelling. A private physician suspected LCH, prompting referral to our institution for further evaluation. A 4 mm punch biopsy of an abdominal papule demonstrated histiocytes with reniform nuclei in the papillary dermis. Positive immunohistochemical staining for S100, CD1a, and CD207 established the diagnosis of LCH.

Several months later, the patient developed twenty nail dystrophy, accompanied by periungual erythema, edema, and pustule formation. Differential diagnoses included onychomycosis, paronychia, and LCH-associated onychodystrophy. A potassium hydroxide test for fungal elements was negative. Clindamycin reduced the swelling and pustules, but onychodystrophy has persisted until the time of writing.

### Patient no. 10

TF is a 1-year-old female who presented with a 4-month history of pallor, abdominal distension, and bipedal edema, along with a 2-week history of erythematous to violaceous macules and papules on the back, scalp, chest, abdomen, and palms.

Skeletal survey revealed hepatosplenomegaly, osteolytic lesions in the left parietal and occipital regions and right fifth rib, as well as vertebra plana. A 3.5 mm punch biopsy of a back papule revealed a focal area of dense mixed-cell infiltrate composed of mostly reniform histiocytes. Immunohistochemical staining of histiocytes was positive for S100, CD1a, and CD207, confirming the diagnosis of LCH.

## Discussion

**Fig 1:**
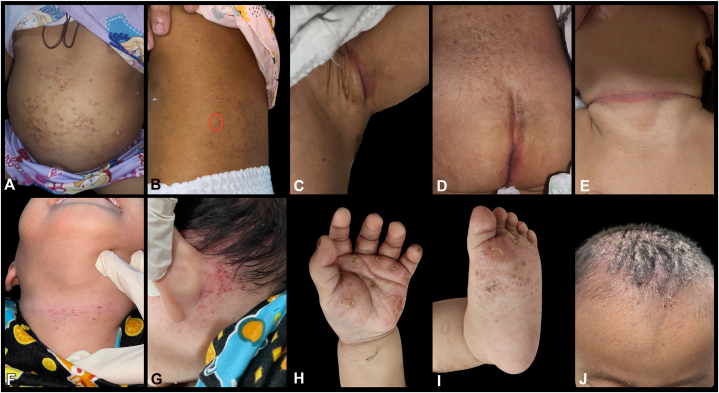
Predilection areas and special sites in Langerhans cell histiocytosis. **A** and **B,** Trunks of patients 9 and 10, with crusted papules and petechiae, respectively. **C** and **D,** Axilla and intergluteal fold with eczematous plaques of patient 2. **E** and **F,** Anterior neck fold of patients 7 and 4 with an eczematous patch and petechiae, respectively. **G,** Retroauricular area with petechiae. **H** and **I,** Palms and soles with crusted brown papules. **J,** Scalp with seborrheic dermatitis-like scales and erythema of patient 7.

In children under 2 years of age, cutaneous manifestations are the most common presenting feature of LCH,^1^ as seen in 8 of ten patients in our cohort. All LCH patients referred to and seen by our Dermatology department had MS disease, most often involving the skin and bones. The time to diagnosis ranged from 2 months to 3 years, though the delay in some cases may also be a reflection of social determinants that resulted in delays in seeking appropriate care. In our center’s experience, a thorough full-body examination is essential when evaluating children for suspected LCH, with particular attention to areas such as the retroauricular regions, genitalia, palmoplantar surfaces, scalp, and nails. Fig 1 illustrates the predilection areas and special sites we inspect in practice for pediatric LCH.

**Fig 2:**
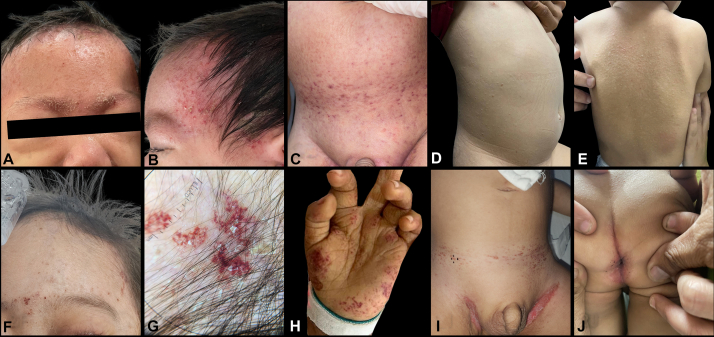
Diverse cutaneous presentations of Langerhans cell histiocytosis. **A,** Seborrheic dermatitis-like lesions appearing as greasy yellow scales and erythema on the glabella and bilateral eyebrows of patient 4. **B** and **C,** Rose-colored papules and petechiae on the face and lower abdomen of patient 4. **D** and **E,** Skin-colored papules on the trunk of patients 5 and 7, respectively. **F** and **G,** Clinical and dermoscopic view of hemorrhagic macules on the face in patient 1. **H,** Hemorrhagic macules on the palm of patient 10. **I** and **J,** Eczematous erosive plaques mimicking napkin dermatitis in patients 2 and 7, respectively.

**Fig 3:**
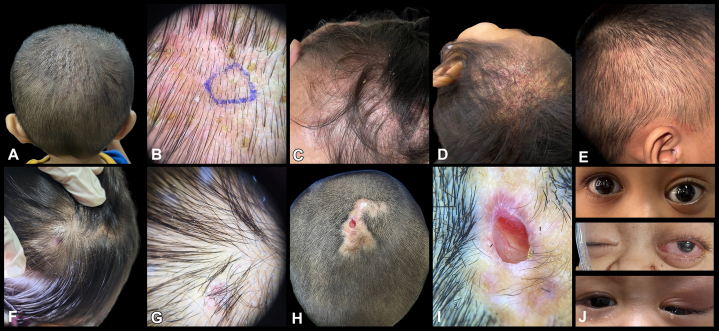
Scalp and orbital changes in Langerhans cell histiocytosis. **A** and **B,** Seborrheic papules with greasy yellow scales. **C,** Scalp erythema and scaling. **D,** Hemorrhagic macules. **E,** Rose-colored papules. **F** and **G,** Solitary scalp nodule. **H** and **I,** Solitary scalp ulcer. **J,** Globe dystopia.

LCH is a known mimicker of common childhood dermatoses. Seborrheic dermatitis-like lesions are most frequently reported in literature, followed by papules, nodules, petechiae, hemorrhagic lesions, eczematous patches, and recurrent diaper dermatitis.^1^^,^^4^, ^5^, ^6^ In the present case series, the majority of cases presented with seborrheic dermatitis-like, hemorrhagic, and/or rose-colored lesions. The scalp, trunk, and intertriginous areas were the most affected sites, consistent with other retrospective cohorts.^4^, ^5^, ^6^ Figs 2 and 3 showcase the spectrum of cutaneous manifestations of LCH, with Fig 3 focusing on craniofacial involvement.

**Fig 4:**
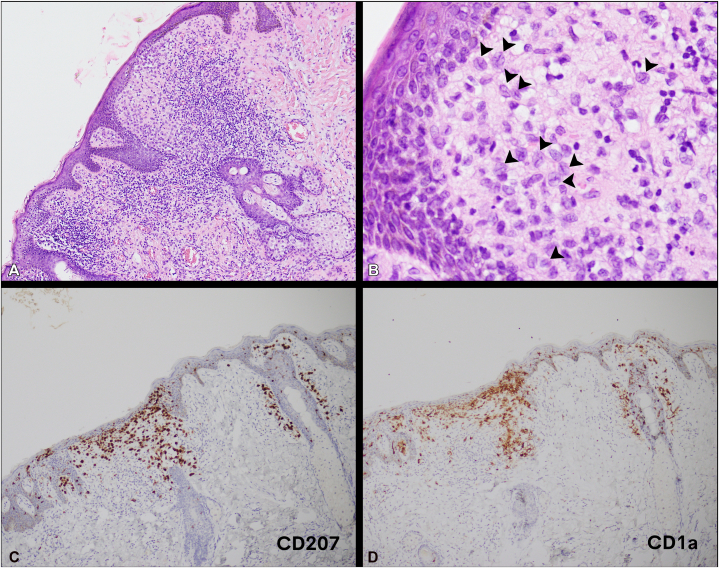
Biopsy findings in Langerhans cell histiocytosis. **A,** Low power view of the scalp wedge biopsy of patient 6 shows vacuolar interface change and dense infiltrates composed of lymphocytes and histiocytes. **B,** High power view reveals clusters of reniform cells in the papillary dermis. **C,** CD207 (Langerin) stains cells of interest in the papillary dermis and epidermis. **D,** CD1a also stains cells of interest.

A retrospective study by Poompuen et al showed delayed diagnosis in patients with seborrheic dermatitis-like lesions and earlier detection in those with petechiae or hemorrhagic findings.^4^ While this trend was not mirrored in our series, such findings can heighten clinical suspicion for LCH and should prompt biopsy and systemic screening. Characteristic histopathology of LCH includes dense dermal infiltrates of cells with reniform or “coffee bean”-shaped nuclei staining positive for S100, CD1a, and CD207 (Langerin), the latter being most specific (see Fig 4).

**Fig 5:**
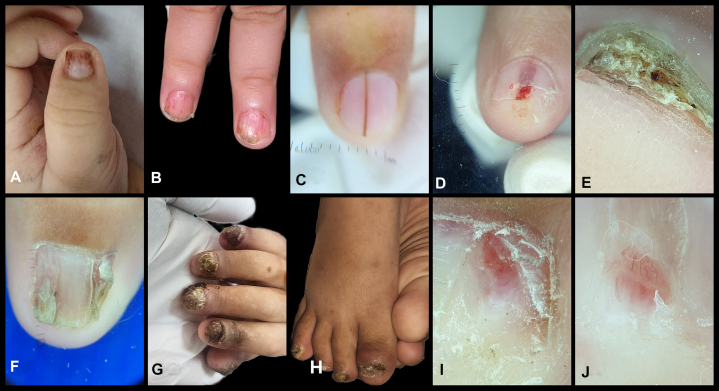
Nail changes in Langerhans cell histiocytosis. **A** and **B,** Splinter hemorrhages. **C,** Longitudinal melanonychia. **D,** Longitudinal hemorrhagic striae mimicking erythronychia. **E,** Subungual hyperkeratosis. **F,** Distal and proximal onycholysis. **G** and **H,** Subungual pustules that resulted in onycholysis and yellow crusting on the nail bed. **I,** Subungual tumor with comma vessel. **J,** Subungual tumor with linear and hairpin vessels.

Despite current observations that nail involvement in LCH is uncommon, 5 out of ten (50%) of our patients had nail findings (see Fig 5). The findings were diverse and included splinter hemorrhages, subungual hemorrhages mimicking longitudinal erythronychia, onycholysis, onychodystrophy, twenty nail dystrophy, subungual hyperkeratosis, pustules, paronychia, and vascular subungual tumors involving multiple digits. In the absence of histopathologic confirmation, however, it remains unclear whether these findings represent true nail unit involvement or nonspecific changes in the setting of systemic disease.

**Fig 6:**
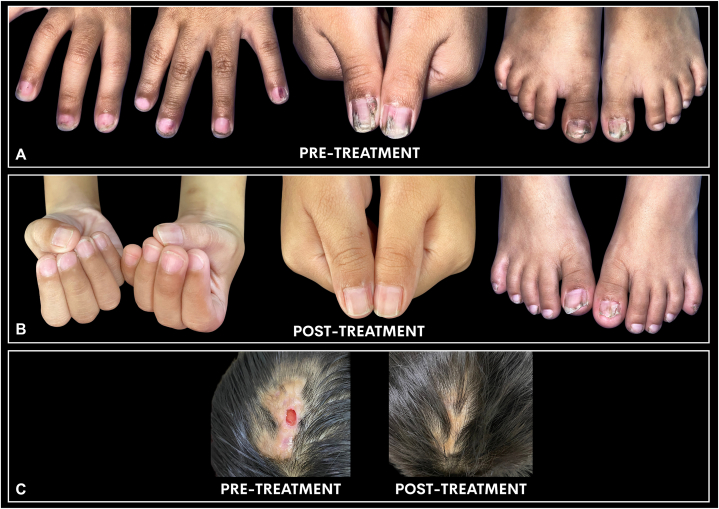
Nail and scalp changes before and after treatment of Langerhans cell histiocytosis. **A,** At baseline, the patient had onycholysis, subungual hemorrhages, subungual hyperkeratosis, and vascular subungual tumors. **B,** After completing 1 year of chemotherapy, onycholysis, subungual hemorrhages, and subungual tumors of the fingernails resolved, while onycholysis of bilateral halluces had partially improved. **C,** Scalp ulcer was therapeutically excised during the biopsy, with no recurrence noted in the interim and significant hair regrowth in the affected area.

Nail involvement is often associated with MS disease, though its prognostic significance remains uncertain.^7^, ^8^, ^9^ In previously reported cases,^7^, ^8^, ^9^ nail findings generally improved following systemic therapy, as seen in patient 6 of our series (Fig 6); as of writing, 5 patients are still undergoing treatment. Further studies evaluating nail changes in LCH with histopathologic confirmation are recommended to help clarify the association of nail findings with LCH. Distinguishing true nail unit LCH from nonspecific nail changes associated with systemic LCH may be challenging in practice, but this distinction may have implications.

It is proposed that the therapeutic response of nails runs an independent course from that of other involved organs and depends on the extent of nail matrix involvement.^7^^,^^9^ As seen in case 6 and previous reports,^9^, ^10^, ^11^, ^12^ nail changes may be the earliest LCH manifestation; majority of pediatric patients in literature also had concomitant cutaneous findings, and one-third had risk organ involvement. Nail evaluation at presentation has dual importance in facilitating early diagnosis and prompting systemic workup.

Reports of LCH presenting as a nonhealing scalp ulcer are scarce. The same patient who presented with nail involvement also presented with a nonhealing scalp ulcer (Fig 6). Gannaban et al described a 2-year-old Filipino child with a refractory scalp abscess, later histopathologically confirmed as LCH.^13^ Ulcers have also been reported in other sites, such as postvaccination arm ulcers in a 21-month-old Chinese female later diagnosed with single-system LCH.^14^

Mucosal lesions, though rare, may present as oral or genital ulcers, gingivitis, bleeding, jaw fractures, or tooth loss.^1^ In this cohort, 1 patient had oral ulcers and another, gingival swelling. Two patients had eczematous plaques in the perianal and intergluteal areas mimicking diaper dermatitis.

Given the heterogeneity of cutaneous morphologies in LCH, we emphasize a vigilant, full-body skin exam and systemic assessment. Patients with cutaneous findings tend to be diagnosed earlier, an emphatic point that the skin is a valuable window to diagnosing LCH. However, challenges persist in atypical cases and those resembling common pediatric dermatoses. Clinicians must hone skills in pattern recognition and maintain a high index of suspicion. Skin biopsy, supported by immunohistochemistry and laboratory and imaging studies, remains essential for diagnosis. When initial biopsies are inconclusive but clinical suspicion remains high, a repeat biopsy should be pursued.

Collaboration between Dermatology and Pediatrics remains integral to timely recognition and management. By emphasizing clinical images and morphology-based clues, this case series aims to sharpen clinical acumen and reinforce the diagnostic value of the skin and nails in uncovering systemic disease. Ultimately, broader familiarity with these features supports earlier detection and improved outcomes in pediatric LCH.

## Conflicts of interest

None disclosed.

## References

[1] Krooks J., Minkov M., Weatherall A.G. (2018). Langerhans cell histiocytosis in children: history, classification, pathobiology, clinical manifestations, and prognosis. J Am Acad Dermatol.

[2] Jezierska M., Stefanowicz J., Romanowicz G., Kosiak W., Lange M. (2018). Langerhans cell histiocytosis in children – a disease with many faces. Recent advances in pathogenesis, diagnostic examinations and treatment. Postepy Dermatol Alergol.

[3] Haupt R., Minkov M., Astigarraga I. (2013). Langerhans cell histiocytosis (LCH): guidelines for diagnosis, clinical work-up, and treatment for patients till the age of 18 years. Pediatr Blood Cancer.

[4] Poompuen S., Chaiyarit J., Techasatian L. (2019). Diverse cutaneous manifestation of Langerhans cell histiocytosis: a 10-year retrospective cohort study. Eur J Pediatr.

[5] Ren F.L., Skipper D.C., Elbendary A., Tan Q., Elston D.M. (2020). Cutaneous manifestations of Langerhans cell histiocytosis in children: a retrospective cohort study of 43 patients. J Eur Acad Dermatol Venereol.

[6] Dhar S., Srinivas S.M., Dhar S. (2020). Langerhans cell histiocytosis in children: a retrospective case series of 126 cases. Pediatr Dermatol.

[7] Mataix J., Betlloch I., Lucas-Costa A., Pérez-Crespo M., Moscardó-Guilleme C. (2008). Nail changes in Langerhans cell histiocytosis: a possible marker of multisystem disease. Pediatr Dermatol.

[8] Figueras-Nart I., Vicente A., Sánchez-Schmidt J. (2016). Langerhans cell histiocytosis presenting as fingernail changes. JAAD Case Rep.

[9] Bender N.R., Seline A.E., Siegel D.H., Sokumbi O. (2019). Langerhans cell histiocytosis with prominent nail involvement. J Cutan Pathol.

[10] Bonometti A., Passoni E., Finotto S., Berti E. (2021). Nail involvement in Langerhans cell histiocytosis and its association with multisystem presentation and lung involvement. Indian J Dermatol Venereol Leprol.

[11] Prayogo R.L., Rahmayunita G., Agustin T., Sirait S.P., Arisanty R. (2020). Purpuric nail striae as the initial manifestation in multisystem Langerhans cell histiocytosis. Pediatr Dermatol.

[12] James V., Prakash A., Indumathi C. (2021). Twenty nail dystrophy in a child with Langerhans cell histiocytosis and tuberculosis. Sudan J Paediatr.

[13] Gannaban R.A., Calimag P., Zapanta Z. (2011). Langerhans cell histiocystosis presenting as a scalp abscess: a case report. Philipp J Surg Spec.

[14] Wang X.M., Liu Y.Q., Li B., Li M., Peng Y., Jiang W.C. (2024). Single-system Langerhans cell histiocytosis with skin ulcers as the initial presentation. Acta Derm Venereol.

